# Dr. Adams, on Morbid Poisons

**Published:** 1803-04-01

**Authors:** Joseph Adams

**Affiliations:** Madeira


### To Dr. WILLAN.

MY DEAR SIR,

YOU will readily believe that your's of the 12th June, 1800,* did not lessen my anxiety to investigate a morbid poison which, it seems, I was the first to announce to the public, but on no other knowledge than what I derived from Dr. Jenner by means of Mr. Cline. If you ask, why I did not pursue the subject further, before my departure from England, my answer is, that when Mr. Cline, by my request, made his inquiries of Dr. Jenner, we were informed among other particulars, that the Inventor was engaged in experiments to ascertain the laws of the poison. After this, it appeared but justice to leave the whole in Dr. Jenner's hands, at least till he should offer the result of his experiments to the public. For this reason, much against my own inclination, I suppressed part of the information with which that gentleman, by a liberality that has marked every part of his character, favoured me. The probability that the Cow-pox originated in the grease from the horse's heel, and was the parent of the Small-pox, would very much have strengthened my suggestion, that "diseases in one class of animals, when communicated to another, seem to alter many of their properties, and also, that some of the morbid poisons which at present so much shorten the period of human life, might owe their origin to such causes."† But though Dr. Pearson had previously sent me a thread; though we had since received matter on glass from Lisbon, by means of Mr. Murray, our late consul general, and from Mr. Banger, one of our principal merchants; though the latter had actually procured a subject to be inoculated in the Tagus, who, had the insertion succeeded, would have arrived just in time for the maturation of the pustule, yet all our endeavours proved abortive. Nor were we more successful with matter from the Vaccine Institution in London, nor with some from Dr. Jenner, left us by different practitioners in their way to

* "The Cow-pox still keeps its ground, not less than 1400 persons having been inoculated without any material suffering, and without taking the Small-pox since. You will be pleased with seeing the progress of this singular complaint, which illustrates your ideas even better than the Small-pox." *Extract of a Letter from Dr. Willan to Dr. Adams.*

† See Morbid Poisons, p. 155 and 156.

the East and West Indies. What added greatly to our disappointment was, that the Small-pox, from which we had been free for ten years past, now visited the island with such fatality, that many parents, who had hitherto rejected every proposal for inoculation, were anxious to avail themselves of it.

Such a calamity called for every means that could be suggested by the Faculty, or enforced by Government, to arrest it. Availing myself, therefore, of the disposition of our Governor, I requested that every means should be made use of to discover whether the disease existed among any of the cows; but after the most diligent inquiry, only a single case occurred, and this by no means characteristic. Trial was however made, but without effect.

At length the arrival of Dr. Gill, in his way to Barbadoes, proved the fortunate event that was to introduce this blessing among us. As we had hitherto all failed in our attempts, Dr. Gill was prevailed on to undertake the management of some dry matter he brought with him; but he was equally unsuccessful. He favoured me with a glass; and as there are some circumstances which hitherto have not occurred, or at least have not been observed, I shall take the liberty of being very particular in my narration. And first, it is necessary that I should describe the scene of my experiments.

From E. N. E. of this town, we have the easiest ascent to those mountains which divide us from the north side of the island; on this account it was probably our earliest road. About a league from Funchall is a small district called *Palheiro do Ferreiro*, or the Blacksmith's Shed, probably because at this place a forge had been erected, to reinstate such shoes as had been cast in this short journey from the town over an ill paved road. Though the improvement of the country has rendered this resource less necessary, yet the remains of the shed existed in the memory of some people still alive, and a clump of chesnut and walnut-trees, now grown to a venerable size, serve to mark the spot, and perpetuate, if not the name, at least the trade of the planter. From this clump you have a view of the sea, by an opening between a high hill and the northern mountains. The prospect is rendered more picturesque by the islets called *Desertos* presenting themselves, and because from the elevated spot you stand on, the sea is visible beyond the high rocks of which those islets consist. Occasionally also the sight of a convoy or single vessels enlivens the view, and awakes the mind from from the contemplation of still life by the expectation of letters or intelligence from Europe; for by this route they should always pass for the Bay of Funchall.

It is not to be wondered if Mr. De Carvalhal, the gentleman of the first landed property in the island, and whom you probably met with during his seven years residence in England, should fix on such a spot for his country residence. Here he has built a pavilion in the modern English style; and having the advantage of more level ground than the country usually affords at such an elevation, has availed himself of the climate to form a retreat much resembling the description of Cyrus's garden, as given us by Zenophon. You will readily conceive the influence which well-directed wealth must produce in such a neighbourhood, every individual of which is growing richer and happier, by being constantly employed when in health, and assisted under illness or any other distress.

Fortunately this was the only place near the town that had not been visited by the Small-pox. For this and other reasons, it was the most desirable place for introducing a medical improvement like the Cow-pox. The recommendation of the *Morgado* (a term similar to that of Laird in Scotland) was sufficient to make the practice popular among the tenantry, especially as the little loss of time that might attend the bringing of their children was always compensated to the parents.

All our attempts had hitherto proved ineffectual; having, therefore, thought long on the subject, I determined to avoid all those inconveniences to which our former ill success might be imputed. Four young subjects were selected, on each of whom two punctures were made. After this the tip of a small camel's hair pencil was dipped in water, and the vaccine matter worked with it in the same manner as painters work their water colours. By the time a complete solution was effected, the little punctures ceased to bleed, and what blood had collected was carefully wiped off; the orifices were then *painted* over, and the usual caution observed to leave them to dry.

On the fourth day I renewed my visit to the *Palheiro do Ferreiro*,* and received the usual account that none of the insertions had succeeded. As I could not myself find out the different cottages, and as all my former examinations

E e 2

* Since called by the governor, on the first visit he made to that place, *Bem fico.*

tions had made me familiar with this intelligence, I was easily satisfied with the accounts of others.

In this state things remained till Sept. 17, an exact lunar month from the day of inoculation, when I received a letter from Mr. Carvalhal, to inform me that we had at last succeeded. At the same time, Mr. Eeles, an English gentleman on a visit at the house, wrote me word that the subjects inoculated might furnish matter for the whole island. You will easily conceive I lost no time in going, fearful lest the pustule should be broken or become dry. On my arrival the patient was sent for, and I leave you to guess my astonishment at the sight of a boy completely covered with Small-pox pustules. Such were my first impressions, and such my amazement, that I remained for some time motionless, till roused from my reverie by a general burst of laughter from my surrounding friends. Though awakened from my first surprize, I could not heartily join in the laugh. After satisfactory assurances that this was among the subjects inoculated, I examined the arm, but could find nothing to distinguish the inoculated part. I next proceeded to dissect the pustules, as I have called them, but found them mere vesicles filled with transparent lymph. On searching for the slough which should have lined the *foveolus*, I found the bottom red, and on a level with the *rete mucosum*, the cavity being entirely made up by the elevation of the cuticle. On inquiry, it appeared that the present was the seventh day from the commencement of the fever, and the fourth of the eruption. I ought to have observed, that the pale vesicular appearance was sufficiently distinguishable on the first view from the usual appearance of so large a crop of Small-pox; but it was not at all unlike that species which Mead calls the chrystalline. Whenever you meet with these again, I wish you would search for the slough.

All that remained to satisfy every remaining doubt was to enquire, whether there was a chance of any previous exposure to variolous infection; to try the effects of inoculation from this subject; and, lastly, to allow an uninterrupted intercourse with the neighbouring cottagers at home and in the field. Two subjects were brought for inoculation, in one of which I discovered a cicatrix which led me to suspect he had been inoculated at the same time as my eruptive patient. Such I was informed was actually the case, which was confirmed by the event of two subsequent inoculations with recent matter; so that he must have have gone through the process with so little inconvenience, that the whole was entirely overlooked.

From this time my *eruptive* patient, having no fever, and finding less inconvenience from the eruption than might be expected, continued with his family and the neighbouring cottagers, without either restriction or medicine. In about a fortnight's time, a child at the breast appeared still fuller of a similar eruption; the face was completely covered, but without any confluence. Satisfied that the former case must be Vaccine, I conceived I had now made a discovery that the disease, under a full eruption, might be infectious. I was however informed, that this was among my first inoculated subjects; and on examining the arm, there appeared a vesicle somewhat larger than the rest, though without the indentation that usually marks the inoculated spot; it was in a similar stage with the others, towards maturation. The vesicles, in whatever part of the body they were examined, appeared as in the former case, red at the bottom, without slough, and the fluid limpid. In both subjects, as the vesicles dried, they rarely left scabs, the new cuticle being formed under the old one, as in a common blister, where the dead skin, as it is called, is preserved.

I was now in daily expectation of seeing other children casually infected; but as both these had been inoculated, the event was still doubtful. Several subjects were inoculated from the last, in succession, in order to preserve the recent matter; but only one had a few vesicles, which appeared after the exciccation of the insertion. The second eruptive case went through the disease without a single untoward symptom, without medicines, and without caution. Being, as before remarked, at the breast, she was constantly in arms; and when abroad, under the woman's woollen cloak: when at home, confined to a small cabin, intended in this mild climate only as a shelter from rain, or retirement for sleep. Under all these disadvantages I watched eagerly for the eleventh day, which passed off without any secondary fever. In a few days after, the mother made a pilgrimage with her child to St. Antonio da Serra, to fulfil a vow made during the illness. This is an undertaking which few women but natives would have ventured upon, even if unincumbered; for though the distance is not more than two leagues, yet from the nature of the country, it could not be accomplished in less than twelve hours. As our rainy season had now approached, and as those high hills, even in the summer, continually attract the clouds, you will not wonder if she was retarded in her journey. Her small stock of provision was soon exhausted, and there were no means of procuring more. You will easily judge what must be the state of the poor convalescent infant. However, they survived this pious journey; the woman preserved her milk, but the child afterwards proved anasarcous. By a little bark for the infant, and the assistance of a more generous diet from the pavilion for the mother, both soon recovered, and are now in perfect health.

Such is the history of these cases. Let us now attend to the inferences which may be drawn towards ascertaining the laws of Vaccination. After a close inquiry concerning the two subjects, I was convinced there had been less intercourse between them than most of the other cottagers and their children. The boy being a cow-herd, was constantly in the fields during the day, and when he returned at night slept in a cot with a child who never received the infection. The infant, according to the custom of the country, was constantly with its mother, who spent her time with her neighbours at the door of their cottages, or of her own. In these parties the children always mix; and such is the general intercourse, that an infectious disease never fails to spread among them. But though the inoculation was continued only in classes, in order to perpetuate the recent lymph, yet none were casually infected, and the inoculation produced universally such mild symptoms, that the mothers could hardly believe their children would be thus easily *sempre livros da bexigas*, for ever free from the small-pox. How then should these appearances occur, so different from what has hitherto been remarked? The following is the only rational solution I can offer.

My reason for being so particular in describing Mr. De Carvalhal's house, was, that you might be aware how directly it is exposed to every breeze from the south-east. Those hot winds called in Africa, Harmattans; in the Mediterranean, Levanters; and in Italy, the Sirochs; occasionally visit this island, which is I believe the westernmost point of land at which they ever arrive. I need hardly refer you to Lynd or Norris for an account of the Harmattan, to Brydone for the Siroch, your own acquaintance among such as have visited the Mediterranean, will refresh your memory sufficiently. It is enough for me to give you some account of what we call a *Leste* in this country.

Whenever the wind blows from the South East, we have for for the most part warm and dry weather, but at certain periods there is a kind of gloomy serenity in the air. The sun seems less powerful, being obscured by a general haziness in the atmosphere, yet the heat is much greater; and such is the universal dryness, that our mahogany card tables are warped, delicate pieces of inlaying fall out, and sheets of veneering, which cover larger furniture, blister and break off. The mucus of the eye is so soon exhausted that a painful kind of stiffness is felt, accompanied with a sensation like scratching, whenever the eye-lids are brought over in order to restore the necessary moisture. Most, perhaps I might say all, of the hygrometers in the island, constructed on the principle proposed by Doctor Franklin, have become useless, probably by never recovering from the injury they sustained during these winds. Those I received from Mr. Lane, are too limited in their scale to show any thing like the dryness of these winds.

You will readily comprehend, that though the valley in which Funchall is situated, is warmer than most other parts of the island which enjoy the benefit of the sea-breeze, yet as we derive this advantage from being sheltered from every wind but the South; so when a *leste* prevails, it is somewhat tempered by the interruption of our eastern hills. Hence *Palheiro do Ferreiro* being the first high lands exposed to these currents, receives its full shock. At the time the subjects were inoculated, we had a more violent and longer continued *leste* than I have ever experienced. The natives complained more of it than the English, and were apprehensive of very serious consequences to their vines; many of these suffered exceedingly, and even some of the oranges and hardier chesnut trees were considerably blighted.

If the *leste* continues long, the thermometer hovers about 80°, rarely exceeding it in the middle of the day, and seldom falling much lower early in the morning. This heat might be tolerable, if we derived any advantage from our breezes; but at these times the air itself conveys the fuel. In the town it seems a little tempered by our Inbat from its passage through the Gullies; but at the Palheiro do Ferreiro they have not even this relief; and at the time I am speaking of, the air absolutely seemed to scorch.

In the midst of all these inconvenencies, some advantages are derived from the *lestes.* Invalids who resort hither, particularly for pulmonary complaints, find a relief which so animates them, that they usually express a wish for a perpetual *leste*. But this leads me to Norris's account of these winds in Africa, part of which I shall transcribe.

"So far, says our author, its effects on the animal and vegetable world are very disagreeable; but it is also productive of some good. The state of the air is extremely conductive to health, it contributes surprizingly to the cure of old ulcers and cutaneous eruptions. Persons labouring under fluxes and intermittent fevers generally recover in a Harmattan; and they who have been weakned and relaxed by fevers, and sinking under evacuations for the cure of them, particularly bleeding (which is often injudiciously repeated) have their lives saved in spite of the doctors. It stops the progress of epidemic diseases: the small-pox, fluxes, and remittent fevers, not only disappear, but they who are labouring under them are almost sure of a speedy recovery. Infection is not then easily communicated. In the year 1770, I had above 300 slaves on board a ship in the Whydah Roads, when the small-pox appeared among them. The greater part of these were inoculated before an Harmattan came on, and about 70 underwent that operation after it sat in: the former got very well through the disorder; none of the latter had either sickness or eruption. We thought we had got clear of the disorder, but in a few weeks it began to appear among these seventy. About fifty of them were inoculated a second time; the others had it in *the natural way*. An Harmattan came on, and they all recovered, except one girl, who had a malignant ulcer on the inoculated spot, and afterwards died of lock jaw."*

After the above statement, it surely implies no disposition to theory, should we admit that in those subjects on whom the Cow-pox appeared over the whole body so long after all expectation of infection had been given up, the local infection had been superceded by the *leste*. That afterwards when, to use the very words of our author, "we thought we had got clear of the disease, it should appear in the natural way." Out of seventy of his cases, twenty took the disease with all the symptoms of the natural small-pox. It is a fair inference, though not at all necessary to our purpose, that in consequence of this appearance, the other fifty were inoculated: if so, it is by no means certain that they would not also have shown the

* See Norris's Memoirs of the reign of Bossa Ahadee, king of Dahomy. Also Literary Mag. Vol. III. p. 254.

the disease in the natural way, had it not been superceded by inoculation.

If these premises are admitted, and till you point them out I can see no objections, we may draw the most important inference; namely, that the Cow-pox, in its worst state, without the assistance of inoculation, appears not to be infectious, is not dangerous, and alters none of its properties when renewed by inoculation. If after this, a few solitary instances of suspicious infection should occur, they can have no influence in lessening our opinion of the importance of the discovery, or our gratitude to the Discoverer.

Though I had got over my difficulties so far, they were not to end here. Having inoculated all who had not previously received the Small-pox near Palheiro do Ferreiro, my next business was to bring the infection to town. In this I had no difficulty, as the Morgado sent me a child for that purpose on whom the lymph was recent, and I had previously procured a subject. But the Small-pox had been so universal, that no individual could be procured, excepting those few who had been born since that disease had ceased, among us. In order, therefore, to secure a succession of fresh matter, I obtained of our Governor permission to inoculate all such exposed children as are supported by government. Unfortunately, those in the town had previously taken the Small-pox; and of such who were brought from the country, I could receive no satisfactory accounts.

It is unnecessary to particularise all my cases in the town. Suffice it to say, they were all children at the breast; that none of them had alarming symptoms, or took medicines; that only two had secondary vesicles; that in one of the last, the symptoms did not appear till twenty days after inoculation, yet this case exhibited the most beautiful specimen of the disease of any, showing a circle not less than an inch and a half in diameter, as exact as if drawn by a compass and shaded by a pencil. The secondary vesicles did not appear till the centre of the tumour had scabbed. From the secondary vesicles two children were inoculated; one of whom went through the disease without fever, and with no eruption but from the arm; the other had secondary vesicles. From these, five others were inoculated, none of whom had secondary symptoms, and scarcely fever.

But in the midst of all my successes, I was alarmed by a piece of intelligence which seemed to put all myformer theory at defiance. In the family of the second infant inoculated inoculated in the town, and on whom secondary vesicles appeared, I was informed that a child belonging to the wet nurse had caught the Small-pox of his foster brother, and was full all over. You will suppose I lost no time in examining the truth of this story. In my way, however, I was willing to make large allowances for the imperfect accounts of uninformed people, suspecting I might find a cutaneous eruption very little resembling the Small or Cow-pox. Even admitting the account true, as to the disease, it seemed easy to suppose that the child might have brought the Small-pox from the country; or, having arrived fresh in town, might have felt the effects of a still tainted atmosphere, to which an infant who first breathed in it might be insensible. However, the sight of the patient precluded all doubts. The form, complexion, and even dissection of the eruptions would omit no question. The vesicles were even broader and flatter than in either of the preceding cases. Without therefore attempting any theory, I satisfied myself with making inquiries as minute as possible. Whilst I was thus engaged, a shrewd Portuguese gentlemen arrived, and laughing heartily at my surprise, addressed me thus:

"We have," said he, "an old Spanish proverb, which asserts, that dirty water will wash clean. Know then, my good Doctor, that during the whole time the child you inoculated was under the disease, his whole body was washed every day in cold water. If this was wrong you are answerable for it, as you not only refused us any physic, but desired us to make no alteration from our usual mode of treating the child. However, you are not answerable for what followed, because you could not suspect that as soon as the patient was washed, the nurse made use of the same water and the same hand, unwiped, to wash her own child; and, by continuing this process every day, produced an universal inoculation; for though we are so fond of washing our infants, we do not think it at all necessary to wipe them afterwards."

By this practice you will easily conceive that the skin of young children frequently chaps, as we find the case with those whose hands are often wetted without being well wiped. But even supposing the skin sound, which is rarely the case with the infants of poor people in this place, I see no reason why lymph, thus diluted, should not produce all the effects above-mentioned; and if so, may not a strong sensible perspiration, under particular circumstances, come so loaded with virulent particles as to to affect the skin of a person sleeping in the same bed, without the ordinary inlets to infection, I mean by the lungs?

That matter, when very much diluted, still retains its virulence, is proved by every inoculation with dried lymph; and that the broken skin is not necessary to produce the effect, is evident in most cases of what is called casual inoculation from the cow or horse. Dr. Jenner remarks, in a note on his fifth case,* that, "when the Cow-pox has prevailed in a dairy, it has often been communicated to those who have not milked the cows by the handle of the milk pail."

The patient, whose case he describes, had many of the Cow-pox sores by handling the utensils used by the servants at that time infected. These sores were communicated to her nose, which became inflamed and very much swoln.

But to proceed with our case. I do not remember ever to have seen so melancholy a spectacle in every part of the body, excepting the face, even in the Small-pox. Whether this arose from all the vesicles being primary ones I will not pretend to say. However, they had all much the same appearance. Those on the face dried as readily as those on the body; we had no secondary fever, no salivation, no diarrhœa, no remedies were administered.

The event proved no interruption to the progress of Vaccination, the reputation of which is so well established, that nothing but a want of subjects will prevent its perpetuation. To assist my endeavours in this respect, I have had every assistance I could wish for from government, and the most respectable among the Portuguese, as well as our vice-consul, Mr. Cock. I have already mentioned some of the services derived from the former, but gratitude obliges me thus publicly to acknowledge the attention of our Governor to the welfare of his province, and his liberality to a stranger.

In the midst of my pursuits, I was interrupted, in consequence of having agreed to part with my house to the owners, and disappointed in my expectations of another. A small house would not answer my purposes, and it was too late to set about repairing a large one. Under these circumstances

* Page 41, first Edit.

circumstances, his Excellency Don Jose de Camera, &c. used all the influence that became him to release me from my engagement; and when he could not do it, consistently with his love of justice, gave me my choice of apartments in the Jesuits College, that I might no longer be interrupted in what he conceived a national pursuit. Those gentlemen were so universally celebrated for the care they took to be well accommodated, that it is unnecessary to add how well we are lodged, or how agreeably situated; conveniences I enjoy with the greater pleasure, now that I have this opportunity of publicly acknowledging them.

Adieu, &c.

*Madeira, Dec.* 30, 1802. JOSEPH ADAMS.

